# A clinico-genomic analysis of soft tissue sarcoma patients reveals CDKN2A deletion as a biomarker for poor prognosis

**DOI:** 10.1186/s13569-019-0122-5

**Published:** 2019-09-11

**Authors:** Nam Q. Bui, Joanna Przybyl, Sally E. Trabucco, Garrett Frampton, Trevor Hastie, Matt van de Rijn, Kristen N. Ganjoo

**Affiliations:** 1 Department of Medicine (Oncology), Stanford University School of Medicine, 875 Blake Wilbur Drive, Stanford, CA 94305 USA; 2 Department of Pathology, Stanford University School of Medicine, Stanford, CA USA; 3 Foundation Medicine, Cambridge, MA USA; 4 Department of Statistics, Stanford University, Stanford, CA USA

**Keywords:** Soft tissue sarcoma, Genomics, Prognostic markers, CDKN2A

## Abstract

**Background:**

Sarcomas are a rare, heterogeneous group of tumors with variable tendencies for aggressive behavior. Molecular markers for prognosis are needed to risk stratify patients and identify those who might benefit from more intensive therapeutic strategies.

**Patients and methods:**

We analyzed somatic tumor genomic profiles and clinical outcomes of 152 soft tissue (STS) and bone sarcoma (BS) patients sequenced at Stanford Cancer Institute as well as 206 STS patients from The Cancer Genome Atlas. Genomic profiles of 7733 STS from the Foundation Medicine database were used to assess the frequency of *CDKN2A* alterations in histological subtypes of sarcoma.

**Results:**

Compared to all other tumor types, sarcomas were found to carry the highest relative percentage of gene amplifications/deletions/fusions and the lowest average mutation count. The most commonly altered genes in STS were *TP53* (47%), *CDKN2A* (22%), *RB1* (22%), *NF1* (11%), and *ATRX* (11%). When all genomic alterations were tested for prognostic significance in the specific Stanford cohort of localized STS, only *CDKN2A* alterations correlated significantly with prognosis, with a hazard ratio (HR) of 2.83 for overall survival (p = 0.017). These findings were validated in the TCGA dataset where *CDKN2A* altered patients had significantly worse overall survival with a HR of 2.7 (p = 0.002). Analysis of 7733 STS patients from Foundation One showed high prevalence of *CDKN2A* alterations in malignant peripheral nerve sheath tumors, myxofibrosarcomas, and undifferentiated pleomorphic sarcomas.

**Conclusion:**

Our clinico-genomic profiling of STS shows that *CDKN2A* deletion was the most prevalent DNA copy number aberration and was associated with poor prognosis.

## Background

Sarcomas are rare, heterogeneous mesenchymal tumors with variable tendencies for aggressive behavior. The most important clinical risk factors for recurrence are tumor size, grade, and histology [1] with high risk tumors being classified as size > 5 cm, FNCLCC grade II–III, and an aggressive histology (ex. undifferentiated pleomorphic sarcoma, dedifferentiated liposarcoma, leiomyosarcoma, etc.). However, even among high risk localized sarcomas, there are considerable differences in clinical outcomes with approximately half of patients achieving a long term remission, while half relapse within 5 years [2]. Attempts to decrease the rate of disease recurrence with adjuvant chemotherapy demonstrated either negative [3, 4] or marginally positive results [2, 5]. New prognostic stratification markers are needed to help identify patients at risk of recurrence and possibly apply more intensive or novel treatments in this cohort.

Cancer genomics is playing an increasingly vital role in prognostic stratification of cancer patients. Prominent examples include karyotype analysis in leukemia [6], RNA transcriptome analysis in breast cancer [7], DNA methylation analysis in glioblastoma [8], and DNA mutations in head and neck cancer [9]. Next generation sequencing has entered into mainstream clinical practice with increasing adoption for metastatic cancer patients and recent FDA approval and subsequent Medicare coverage of a sequencing companion diagnostic [10]. With the proliferation of sequencing information, there is opportunity to discover new prognostic correlations from mutational data, especially for rare tumors with limited prognostic features.

In this study, we examined 152 sarcoma patients treated at Stanford Cancer Institute by tumor genome sequencing and explored associations between genomic alterations and outcomes. We also analyzed data from The Cancer Genome Atlas (TCGA) sarcoma project, to independently validate findings from the Stanford cohort. In addition, we explored the Foundation Medicine sequencing database to describe the landscape of *CDKN2A* alterations in STS.

## Methods

### Patient Selection

Between 2012 and 2017, 1291 patients at Stanford Cancer Institute had tumor genomic sequencing performed with hybrid capture based next generation sequencing (Foundation Medicine, Cambridge, MA) [11, 12]. One hundred fifty-two of these patients had soft tissue or bone sarcoma. Patient data including demographics and clinical data were abstracted retrospectively from the medical chart. Data cut-off was December 19, 2018. This study was approved by the Stanford University institutional review board (IRB).

TCGA DNA point mutation and copy number data from 206 sarcoma patients were analyzed through cBioPortal (accessed on 02/19/2019) [13]. In addition, we analyzed the *CDKN2A* mutation status including DNA copy number changes, genomic rearrangements, and SNVs (somatic and germline [14]) in 7733 soft tissue sarcoma patients analyzed by Foundation Medicine. These samples were sequenced as part of routine clinical care following previously described methods [11, 12]. Samples were submitted to a CLIA-certified, New York State-accredited, and CAP-accredited laboratory (Foundation Medicine) for hybrid capture followed by next-generation sequencing using the FoundationOneHeme^®^, FoundationOne^®^, or FoundationOneCDx^®^ platforms.

### Statistical analysis

The Cox proportional hazards model was used for comparative survival analysis. Overall survival was defined as time from diagnosis until patient death. Time to recurrence was defined as time from diagnosis until local or distant recurrence. Time to treatment failure was defined as time from the first day of therapy until radiographic/clinical progression or death. p values < 0.05 were considered significant. In the TCGA survival analysis, associated risk factors for prognosis were determined by running multiple univariate Cox proportional hazards models for age, FNCLCC grade, stage, tumor size, histology, and *CDKN2A* status. Of those, only age, stage, tumor size, and *CDKN2A* status significantly affected prognosis and were thus incorporated into the final multivariate Cox model. Tumor size violated the proportional hazards assumption and therefore a time-transforming function (tt) was used. Statistical analysis was performed in R (version 3.3.2) [15] with the *survival* package (Version 2.41.3) [16].

## Results

### Patient characteristics

Characteristics of 152 sarcoma patients treated at the Stanford Cancer Institute are summarized in Table 1. The average age of the patients was 54.5, ranging from 15 to 90 years old. The vast majority of tumors were soft tissue sarcoma (n = 134) with a small number of primary bone sarcomas (n = 12). The most common tumor type was leiomyosarcoma (LMS) (n = 25), followed by undifferentiated pleomorphic sarcoma (UPS) (n = 23), myxofibrosarcoma (MFS) (n = 13), liposarcoma (LPS) (n = 13: 11 dedifferentiated LPS, 1 myxoid LPS, 1 pleomorphic LPS), and malignant peripheral nerve sheath tumor (MPNST) (n = 9). Most patients had localized sarcoma at diagnosis (70%, n = 106), although almost all patients were diagnosed with or developed metastases during the course of their disease (89%, n = 136). This high rate of metastasis was most likely due to selection bias as genomic sequencing was almost only performed for potential therapeutic options in refractory patients. The extremity was the most common site of disease (30%, n = 46), followed by pelvic (20%, n = 31), and trunk (10%, n = 15). Adjuvant chemotherapy was administered to 41% (n = 48) of patients and adjuvant radiotherapy was given to 66% (n = 78) of patients.

**Table 1:** Demographics of patients treated at Stanford Cancer Institute and TCGA

Characteristic	Stanford patients (n = 152)	TCGA (n = 206)
Age at diagnosis	54.5 (15–90)	60 (20–90)
Sex		
Male	74 (48.7%)	94 (46%)
Female	78 (51.3%)	112 (54%)
Race or ethnic group		
Caucasian	104 (68%)	N/A
Hispanic	22 (14%)	
Asian	22 (14%)	
Other	4 (3%)	
Tumor histology		
Leiomyosarcoma	25	80
Undifferentiated pleomorphic sarcoma	23	44
Sarcoma (NOS)	14	
Myxofibrosarcoma	13	17
Liposarcoma	13	50
Malignant peripheral nerve sheath tumor	9	5
Synovial sarcoma	7	10
Osteosarcoma	5	
Angiosarcoma	5	
Rhabdomyosarcoma	3	
Ewing’s sarcoma	3	
Chondrosarcoma	3	
Malignant phyllodes tumor	3	
Local or metastatic		
Local	106 (70%)	89 (43%)
Locally advanced	8 (5%)	
Metastatic	38 (25%)	46 (22%)
Unknown		71 (34%)
Site		
Extremity	46 (30%)	62 (30%)
Pelvic	31 (20%)	33 (16%)
Trunk	15 (10%)	9 (4%)
Retroperitoneum	21 (14%)	87 (42%)
Spine	9 (6%)	0
Breast	6 (4%)	0
Lungs	4 (3%)	2 (1%)
Other	10 (7%)	13 (6%)
Size (cm)	11.4 (2–42)	12.7 (1.2–39.5)
FNCLCC grade		
I	7 (5%)	14 (7%)
II	23 (15%)	112 (54%)
III	37 (24%)	80 (39%)
N/A	85 (56%)	
Adjuvant chemotherapy		
Yes	48 (41%)	47 (23%)
No	70 (59%)	150 (73%)
N/A		9 (4%)
Adjuvant radiotherapy		
Yes	78 (66%)	58 (28%)
No	37 (31%)	139 (67%)
N/A		9 (4%)

### Soft tissue and bone sarcoma are defined by copy number changes and fusion events

**Fig. 1:**
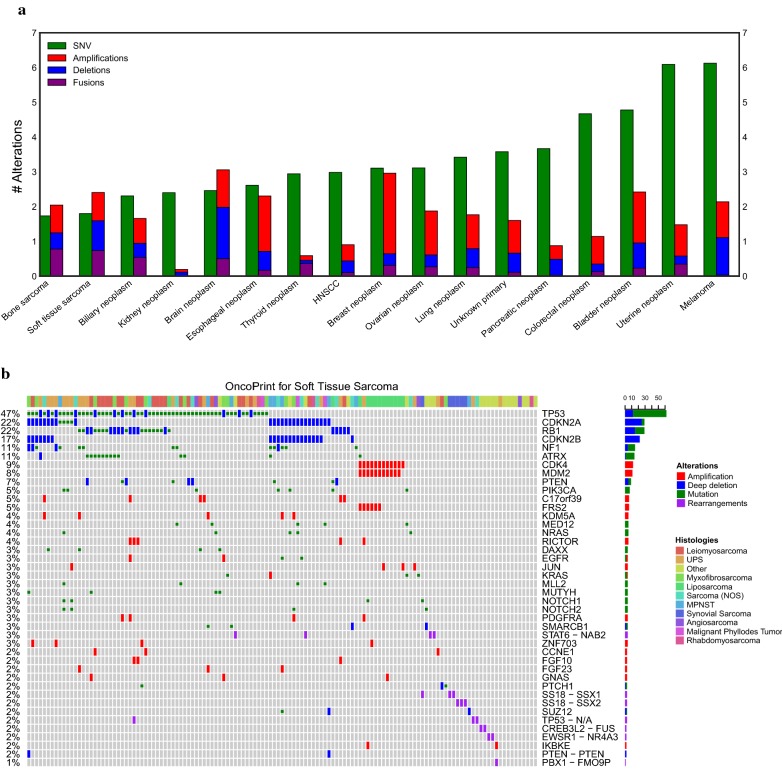
Genomic landscape of bone and soft tissue sarcoma. **a** Average frequency per patient of SNVs, amplifications, deletions, and fusions per cancer type for 1291 Stanford patients with tumor sequencing. When ordered by average number of SNVs, bone and soft tissue have the lowest amount of SNVs while having the highest relative proportion of copy number and structural rearrangements. **b** Oncoprint [30] plot of genomic alterations in soft tissue sarcoma clustered by frequency. The genes are ordered from most frequent (top) to least frequent (bottom). Tumor histology is shown on the topmost colored bar

When compared to other tumor types, the predominant genomic aberrations in soft tissue and bone sarcomas were DNA copy number and chromosomal translocations (Fig. 1a). Based on the cohort of patients treated at Stanford Cancer Institute, sarcomas had the lowest average number of single nucleotide variants (SNVs), averaging 1.7 SNVs per tumor compared to 6.1 SNVs per tumor in melanoma, which had the highest mutation rate among the analyzed tumor types. Conversely, sarcomas had relatively high percentage of copy number and fusion events, representing 57% of all gene alterations compared to 7% for renal neoplasms, which was the tumor type with the lowest frequency of copy number alterations. The number of “targetable” mutations that led to non-standard of care treatment options was low, with only 8 patients receiving targeted therapy (5%). Of these 8 patients, one patient had a complete response (myopericytoma with *NTRK* fusion on larotrectinib study), one patient had stable disease for 9 months (osteosarcoma with *NF2* mutation on everolimus), two patients discontinued treatment due to drug toxicity, and 4 patients progressed rapidly on first follow up scan. The frequency and distribution of genomic alterations across different tumor types is summarized in Fig. 1b. The most frequently affected gene was *TP53* (47%), followed by *CDKN2A* (22%), *RB1* (22%), *NF1* (11%), and *ATRX* (11%). The majority of alterations in *TP53, NF1*, and *ATRX* were point mutations while the predominant alterations in *CDKN2A* and *RB1* were copy number losses. Other than the pathognomonic *MDM2* amplification in liposarcoma, there were no altered genes that clustered into specific histologic types (Fig. 1b, top colored bar). Fusion driven sarcomas (Fig. 1b, purple bar), did not tend to have many co-occurring mutations.

### *CDKN2A* aberrations are associated with poor prognosis in soft tissue sarcoma

**Fig. 2:**
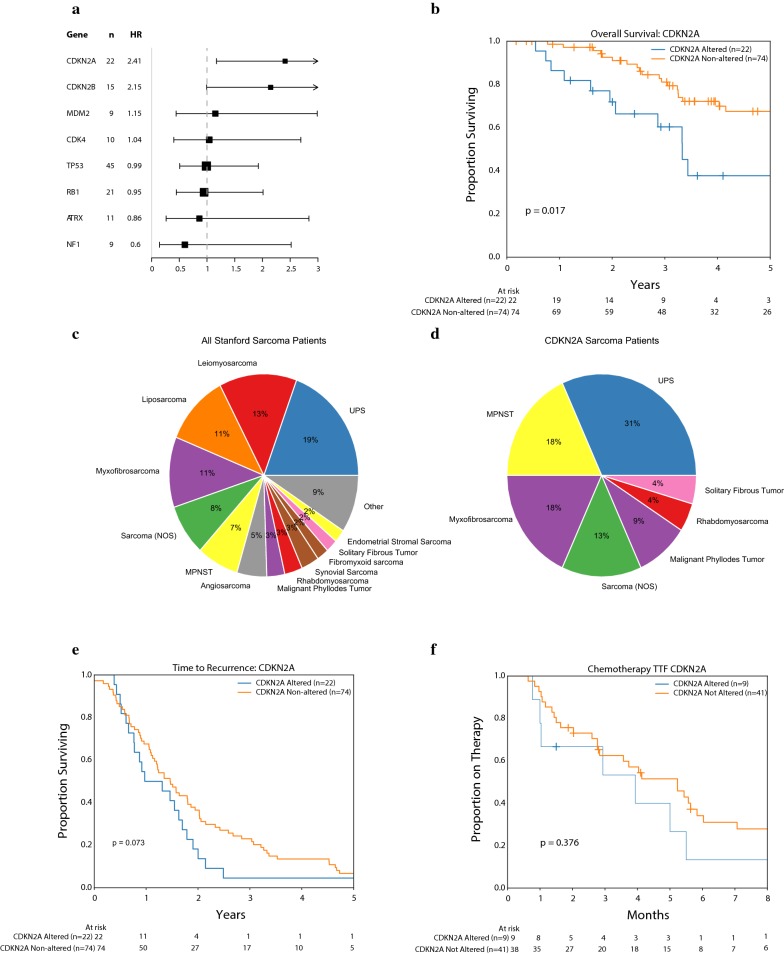
Clinical outcomes for localized soft tissue sarcomas with genomic sequencing treated at Stanford (n = 96). **a** Forest plot of most commonly altered 8 genes with prognosis reveals only *CDKN2A* to be significantly associated with worse prognosis. **b** Kaplan–Meier plot of overall survival for *CDKN2A* altered vs. non-*CDKN2A* altered patients (p = 0.017). **c** Histologic distribution of all patients (**d**) and *CDKN2A* altered patients (C2) reveals increases in representation of MPNST, MFS, and UPS with decreases in LMS and LPS. **e** Kaplan–Meier plot of time to recurrence for *CDKN2A* altered vs. non-*CDKN2A* altered patients (p = 0.09). **f** Kaplan–Meier plot of time to treatment failure for first line systemic chemotherapy for *CDKN2A* altered vs. non-*CDKN2A* altered patients (p = 0.29)

Next, we sought to determine whether the most frequent genomic alterations in sarcoma patients treated at Stanford Cancer Institute correlated with prognosis. In order to standardize the patient population, we selected only patients with STS (excluding GISTs and primary bone sarcomas) and localized disease at diagnosis (n = 96). Of note there were 2 patients with spindle cell histologies originating in bone that were included as they were treated under soft tissue sarcoma protocols. In a Cox proportional hazards model, adjusted for age, only *CDKN2A* alterations were associated with an effect on prognosis with a significantly worse overall survival (OS) (HR 2.83, mOS 3.3 vs. 7.7 years, p = 0.017, Fig. 2a, b). No other genetic alteration was found to significantly associate with survival. Twenty-two patients (23%) had *CDKN2A* alterations of which 18 (82%) were homozygous deletions, 3 (14%) were nonsense mutations, and 1 (5%) was a loss of function SNV. Histological distributions of all sarcoma patients (Fig. 2c) compared with patients with aberrant *CDKN2A* (Fig. 2d), showed an increase in the representation of UPS, MPNST, and MFS patients, and a decrease in the amount of LMS and LPS patients in those with *CDKN2A* loss. Analysis of time to recurrence (either local or metastatic) showed that there was a trend to earlier recurrence with *CDKN2A* altered patients (median 0.87 vs. 1.2 years, logrank p = 0.073, Fig. 2e), however, this was not statistically significant. There was no difference in time to treatment failure (TTF) for first line chemotherapy, with a median time to progression of 3.9 vs. 5.2 months (p = 0.38) for *CDKN2A* altered vs. non-altered patients (Fig. 2f). These results suggest that suggests that there may be an inherently more aggressive biology in patients with alterations in the *CDKN2A* gene, with an earlier time to recurrence from initial surgery, but no substantial increase in resistance to chemotherapy once disease becomes metastatic.

**Fig. 3:**
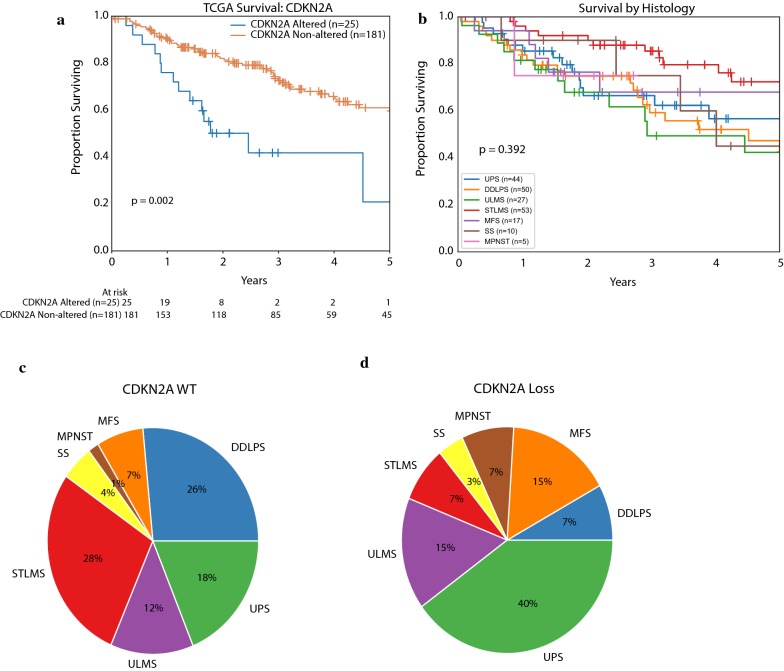
Clinical outcomes for TCGA soft tissue sarcoma patients (n = 206). **a** Kaplan–Meier curve of overall survival for *CDKN2A* altered versus non-*CDKN2A* altered patients (Cox Proportional Hazards adjusted for age, stage, and tumor size, p = 0.002). **b** Kaplan–Meier curve of survival by sarcoma histologies shows no significant difference in survival overall (p = 0.392). **c** Histologic distribution of patients who are *CDKN2A* wild type **c** versus *CDKN2A* altered **d** shows increase in proportion of MPNST, MFS, and UPS with decrease in LMS and DDLPS

We validated these findings in the TCGA sarcoma dataset, which contains DNA copy number and mutation data from 206 primary untreated STS patients and clinical follow up information. The dataset focuses on 6 major sarcoma subtypes including dedifferentiated LPS (DDLPS), LMS, UPS, MFS, MPNST, and synovial sarcoma (SS). There were 25 tumors with *CDKN2A* homozygous deletion, 180 were wild type, and 1 case had *CDKN2A* amplification (TCGA PanCancer Atlas data, accessed on 2/19/2019 through cBioPortal). There was only one patient who had a *CDKN2A* nonsense mutation and this patient also had concurrent *CDKN2A* copy number loss. Among tumors with *CDKN2A* loss there was an increased representation of MPNST, MFS, and UPS patients and a decreased ratio of LMS and LPS, similar to our Stanford data set (Fig. 3c, d). In a multivariate Cox proportional hazards model adjusted for age, stage, and tumor size, *CDKN2A* alterations were significantly associated with a worse prognosis (HR 2.7, mOS 2.5 vs. 6.7 years, p = 0.002, Fig. 3a). Other significant risk factors influencing outcome were age and presence of metastases at diagnosis (Table 2). We also tested other potential risk factors such as tumor histology (Fig. 3b) and grade in univariate Cox models but neither were significantly correlated with survival outcomes. Exploratory analysis of *CDKN2A*-associated prognosis within each histology was performed, albeit at considerable loss of statistical power due to individually small sample size, and demonstrated pronounced survival differences in MFS, STLMS, UPS, and SS (Additional file 1: Figure S1).

**Table 2:** Cox proportional hazards model for TCGA patients

Characteristic	Hazard ratio (95% CI)	p value
CDKN2A alteration	2.7 (1.4–5.1)	0.002*
Unknown stage (reference)	1	
Localized tumor at diagnosis	0.42 (0.23–0.79)	0.007*
Metastasis at diagnosis	1.79 (1.06–3)	0.03*
Age at diagnosis	1.03 (1.01–1.05)	0.002*
Tumor size (cm)	1 (0.99–1.01)	0.053

### The landscape of *CDKN2A* genomic alterations in soft tissue sarcoma

**Fig. 4:**
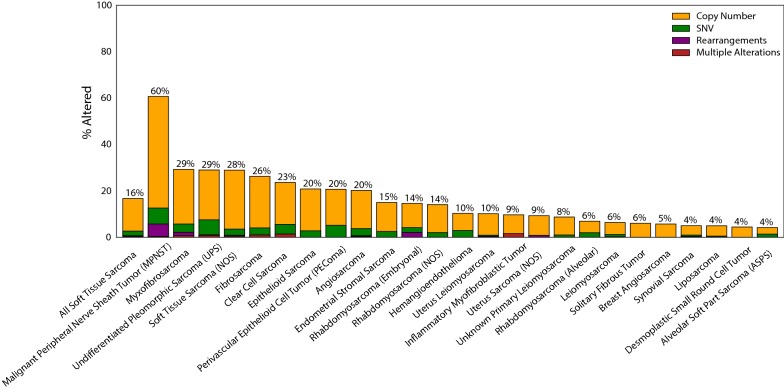
Frequency of *CDKN2A* mutations per histologic sarcoma type in Foundation Medicine dataset (n = 7733). Copy number changes (orange) dominate the mutational landscape with infrequent occurrences of SNVs (green) and rearrangements (purple). The overall prevalence of *CDKN2A* mutations in sarcoma (left most bar) is 16.7% (n = 7733), with the highest mutated tumor being MPNST (60.7%, n = 262) followed by myxofibrosarcoma (29.3%, n = 140), and undifferentiated pleomorphic sarcoma (26.8%, n = 372)

Sarcomas are extremely rare tumors with less than 15,000 new cases diagnosed each year in the United States [17]. Therefore, large, multi-institutional, central databases are critical for analysis of these rare tumors, as it is difficult for a single institution to accrue a sufficiently large patient cohort for comprehensive analysis. We queried the Foundation Medicine database that has sequencing data on 7733 soft tissue sarcomas from numerous institutions for alterations in *CDKN2A*. The frequency of genomic aberrations in *CDKN2A* gene in this multi-institutional cohort was similar to our Stanford cohort, with an alteration rate of 16.7% (Table 3). Almost all of these were copy number changes (loss) at 14.1%, with smaller numbers of SNVs (2.3%), and gene rearrangements (0.5%). There is emerging data on germline *CDKN2A* mutations that predispose towards the development of sarcoma [18], however, we found that these cases were exceedingly rare (0.2%). When broken down into histologic type (Fig. 4), the most commonly affected tumor type was MPNST (60.7%, n = 262), in which *CDKN2A* loss has been shown previously to be a defining event for the malignant transformation of neurofibromas [19, 20]. Other commonly *CDKN2A*-mutated tumors were myxofibrosarcomas (29.3%, n = 140), undifferentiated pleomorphic sarcomas (29%, n = 372), and fibrosarcomas (26.3%, n = 99). Leiomyosarcomas and liposarcomas were found to infrequently have *CDKN2A* aberrations at a ≤ 10% rate. These findings correlate with the histologic distributions both in the Stanford data set as well as the TCGA cohort (Additional file 1: Figure S2).

**Table 3:** Foundation one histology distribution

Disease	n	All CDKN2A	Copy #	Rearrangements	SNV: somatic	SNV: germline	SNV: unknown	SNV: all	Multiple alterations
All soft tissue sarcoma	7733	16.7	14.0	0.4	1.1	0.2	0.7	2.0	0.3
Malignant peripheral nerve sheath tumor (MPNST)	262	60.7	48.1	5.3	5.0	0.4	1.5	6.9	0.4
Myxofibrosarcoma	140	29.3	23.6	1.4	2.1	0.7	0.7	3.6	0.7
Undifferentiated pleomorphic sarcoma (UPS)	372	29	21.5	0.3	5.1	0.0	1.3	6.5	0.8
Soft tissue sarcoma (NOS)	1476	28.9	25.4	0.4	1.3	0.3	1.1	2.6	0.5
Fibrosarcoma	99	26.3	22.2	0.0	2.0	0.0	1.0	3.0	1.0
Clear cell sarcoma	72	23.6	18.1	0.0	0.0	1.4	2.8	4.2	1.4
Epithelioid sarcoma	72	20.8	18.1	0.0	2.8	0.0	0.0	2.8	0.0
Perivascular epithelioid cell tumor (PEComa)	58	20.7	15.5	0.0	1.7	1.7	1.7	5.2	0.0
Angiosarcoma	292	20.2	16.4	0.3	1.7	0.3	1.0	3.1	0.3
Endometrial stromal sarcoma	200	15	12.5	0.0	0.5	0.0	2.0	2.5	0.0
Rhabdomyosarcoma (Embryonal)	96	14.6	10.4	2.1	1.0	0.0	1.0	2.1	0.0
Rhabdomyosarcoma (NOS)	199	14.1	12.1	0.0	2.0	0.0	0.0	2.0	0.0
Hemangioendothelioma	68	10.3	7.4	0.0	2.9	0.0	0.0	2.9	0.0
Uterus leiomyosarcoma	678	10.2	9.3	0.3	0.1	0.1	0.1	0.4	0.1
Inflammatory myofibroblastic tumor	62	9.7	8.1	0.0	0.0	0.0	0.0	0.0	1.6
Uterus sarcoma (NOS)	129	9.3	8.5	0.8	0.0	0.0	0.0	0.0	0.0
Unknown primary leiomyosarcoma	206	8.7	7.8	0.0	0.0	0.0	1.0	1.0	0.0
Rhabdomyosarcoma (Alveolar)	101	6.9	5.0	0.0	0.0	1.0	1.0	2.0	0.0
Leiomyosarcoma	924	6.4	5.2	0.0	0.4	0.2	0.3	1.0	0.2
Solitary fibrous tumor	149	6	6.0	0.0	0.0	0.0	0.0	0.0	0.0
Breast angiosarcoma	70	5.7	5.7	0.0	0.0	0.0	0.0	0.0	0.0
Synovial sarcoma	321	5	4.0	0.0	0.0	0.0	0.6	0.6	0.3
Liposarcoma	805	5	4.5	0.1	0.1	0.1	0.1	0.4	0.0
Desmoplastic small round cell tumor	113	4.4	4.4	0.0	0.0	0.0	0.0	0.0	0.0
Alveolar soft part sarcoma (ASPS)	72	4.2	2.8	0.0	1.4	0.0	0.0	1.4	0.0

## Discussion

In our single institution cohort, we show that *CDKN2A* deletions were the most prevalent DNA copy number aberrations in STS and these aberrations were associated with poor clinical outcome. Based on an independent cohort of patients in the TCGA study, we validated *CDKN2A* loss as an adverse prognostic factor. Finally, we illustrated the landscape of *CDKN2A* loss across a large multi-institutional sequencing database of STS.

Several previous studies have proposed other molecular prognostic markers in sarcoma, however, they have not yet been introduced in the clinical practice. The recent TCGA analysis of STS [21] identified poor performing cohorts of patients within DDLPS and soft tissue LMS. The DDLPS patients with poor clinical outcome were characterized by hypermethylation and certain chromosomal amplifications, while the LMS patients with adverse outcome were characterized by high expression levels of microRNA miR-181b-5p. Other high-throughput studies identified a CINSARC gene expression signature related to mitosis and chromosome integrity that identified high-risk patients and outperformed the histology-based grading system in sarcomas with complex genomic profiles such as LMS, UPS and DDLPS, GISTs, and synovial sarcoma [22, 23]. In regards to the prognostic impact of *CDKN2A* loss in sarcoma, previous work had suggested that its loss might have prognostic significance in Ewing’s sarcoma [24–26], however, a large analysis of 568 Ewing’s patients enrolled on a Children’s Oncology Group (COG) protocol, failed to reproduce this finding [27]. Other work has also found an association between *CDKN2A* deletion and poor prognosis in GIST [22].

*CDKN2A* (cyclin-dependent kinase inhibitor 2A) is a tumor suppressor gene that encodes two proteins: p16 and p14arf [28]. The p16 protein plays a functional role in cell cycle and senescence through the regulation of the cyclin-dependent kinase (CDK) 4/6 and cyclin D complexes. p14arf activates TP53, the canonical tumor suppressor. *CDKN2A* loss of function is seen in a number of different cancer types, with the majority of cases being inactivation by homozygous deletions, followed by less common inactivating mutations and promoter hypermethylation. In our study, we found that loss of *CDKN2A* had a significant correlation with worse prognosis in localized STS at our institution and validated this in an independent cohort of patients from TCGA. The histologic subtypes MPNST, MFS, and UPS had increased frequency of *CDKN2A* loss as compared to LMS and DDLPS.

This data can potentially help further risk stratify patients and inform which patients are at the highest risk of relapse and would warrant adjuvant chemotherapy. This would be especially helpful in STS as the concept of adjuvant therapy is controversial and there is no definitive consensus on whether it should be administered to all patients. Detection of genomic aberrations in *CDKN2A* may also have therapeutic implications since a number of basket and umbrella clinical trials enroll patients with concordant loss of *CDKN2A* and amplifications of *CDK4, CDK6, CCND1, CCND2* and/or *CCND3*. We identified co-existing aberrations in these genes in a small subset (1.5%) of sarcoma patients analyzed in the TCGA study and Memorial Sloan Kettering genomic studies (summarized in Additional file 1: Tables S1, S2). Sarcoma patients carrying these aberrations may be suitable candidates for clinical trials of CDK4 inhibitors.

There are some limitations to this study. First, STS encompass a wide range of natural history, with certain histologies behaving more aggressively, while others behave more indolently. In our single institutional cohort due to the wide variety of tissue types, we were unable to statistically control for tumor type due to anticipated loss of statistical power. However, in the larger TCGA dataset because of the more limited number of histologies, our statistical model accounted for tumor type, and *CDKN2A* was still a significant marker of poor prognosis. Furthermore, in our cohort, genetic sequencing was almost exclusively done on patients that developed advanced disease and thus as a byproduct, acted as a selector for aggressive biology (ex. > 90% of patients developed metastatic disease). Another limitation in our cohort is that sequencing was not done uniformly on the primary tumor at time of diagnosis, thus there could potentially be changes in the genome throughout the disease course that may not be captured by a single timepoint biopsy. In addition, there is the possibility of diverging clones that establish metastatic sites and are genetically distinct from the primary tumor. A potential solution to this is to use circulating tumor DNA (ctDNA) from peripheral blood, which theoretically includes DNA deposits from multiple tumor sites. We have recently shown the clinical utility of detection of SNVs, indels and copy number alterations in ctDNA of patients with LMS, and demonstrated that ctDNA analysis may capture the molecular intra-tumoral heterogeneity of LMS [29]. Another limitation is that in the Foundation One dataset, pathology is not centrally reviewed so the pathologic diagnosis is dependent on the diagnosis term sent in by the referring physician. This can lead to incorrect or outdated diagnoses, as manifested by the high prevalence of fibrosarcoma in the database. Finally, this is a single institutional experience and larger data sets will be beneficial in exploring this association further. In rare tumors, this can be challenging but can be achieved through the work of rare tumor consortiums.

## Conclusions

In summary, we demonstrate the association between genomic aberrations affecting the *CDKN2A* gene and worse prognosis in two independent data sets of STS. We also establish the frequency of *CDKN2A* alterations across histologies in a large STS genomic database, although limited by lack of central review of pathology. Further research is needed into novel therapeutics to target the p16-CDK4-RB1 pathway, and whether additional adjuvant therapy upfront can improve outcomes in this subset of patients.

## Supplementary information

**Additional file 1.** Additional tables and figures.

## Data Availability

The datasets used and/or analysed during the current study are available from the corresponding author on reasonable request.

## Abbreviations

BS: bone sarcoma
CDK: cyclin-dependent kinase
CDKN2A: cyclin-dependent kinase inhibitor 2A
ctDNA: circulating tumor DNA
COG: Children’s Oncology Group
DDLPS: dedifferentiated liposarcoma
FNCLCC: Fédération Nationale des Centres de Lutte Contre Le Cancer
GIST: Gastrointestinal Stromal Tumor
HR: hazard ratio
IRB: Institutional Review Board
LMS: leiomyosarcoma
LPS: liposarcoma
MFS: myxofibrosarcoma
MPNST: Malignant Peripheral Nerve Sheath Tumor
NOS: not otherwise specified
OS: overall survival
SNV: single nucleotide variant
STS: soft tissue sarcoma
TCGA: The Cancer Genome Atlas
TTF: time to treatment failure
UPS: undifferentiated pleomorphic sarcoma
SS: synovial sarcoma
