# Supplementary material for: A clinico-genomic analysis of soft tissue sarcoma patients reveals CDKN2A deletion as a biomarker for poor prognosis
Source: Clin Sarcoma Res. 2019 Sep 11;9:12. doi: 10.1186/s13569-019-0122-5 (PMC6739971; doi:10.1186/s13569-019-0122-5)
Supplement: Supplementary file 1 — Additional file 1. Additional tables and figures. [file 13569_2019_122_MOESM1_ESM.docx]

**Tables:**

**Table S1: Thirteen sarcoma patients with concordant deletion of *CDKN2A* and amplification of *CDK4, CDK6, CCND1, CCND2* or *CCND3* identified based on cBioPortal data from the TCGA PanCancer Atlas analysis, MSKCC/Broad Sarcoma dataset (Nat Genet 2010) and MSK-IMPACT Clinical Sequencing Cohort (Nat Med 2017) accessed on 2/19/2019. (NOS – not otherwise specified, HOMDEL – homozygous deletion, AMP - amplification)**

| **Study** | **Patient ID** | **Diagnosis** | ***CDKN2A*** | ***CDK4*** | ***CDK6*** | ***CCND1*** | ***CCND2*** | ***CCND3*** |
| --- | --- | --- | --- | --- | --- | --- | --- | --- |
| TCGA | TCGA-DX-A3U5 | DDLPS | HOMDEL | AMP |  |  | AMP |  |
| TCGA | TCGA-DX-A8BJ | UPS | HOMDEL | AMP |  |  |  |  |
| TCGA | TCGA-QC-A6FX | UPS | HOMDEL |  | AMP |  |  |  |
| TCGA | TCGA-DX-A1L1 | DDLPS | HOMDEL |  |  | AMP |  |  |
| TCGA | TCGA-QQ-A5VC | LMS | HOMDEL |  |  |  |  | AMP |
| MSK-2010 | PT173PL | Pleomorphic LPS | HOMDEL |  | AMP |  |  | AMP |
| MSK-2010 | PT56GT | Epithelioid GIST | HOMDEL |  |  | AMP |  |  |
| MSK-2010 | PT137MF | MFS | HOMDEL |  |  |  |  | AMP |
| MSK-IMPACT-2017 | P-0006882 | Sarcoma, NOS | HOMDEL | AMP |  |  |  |  |
| MSK-IMPACT-2017 | P-0007111 | DDLPS | HOMDEL | AMP |  |  |  |  |
| MSK-IMPACT-2017 | P-0001217 | UPS | HOMDEL |  | AMP |  |  |  |
| MSK-IMPACT-2017 | P-0007376 | Sarcoma, NOS | HOMDEL |  | AMP |  |  |  |
| MSK-IMPACT-2017 | P-0002757 | Intimal sarcoma | HOMDEL |  |  |  |  | AMP |

**Table S2: Summary of sarcoma patients with co-existing deletion of *CDKN2A*, and amplifications of *CDK4/6* and *CCND1/2/3* genes in three independent datasets.**

|  | **Number of patients with *CDKN2A* homozygous deletion** | **Number of patients with homozygous deletion of *CDKN2A* and amplification of *CDKs* or *CCNDs*** |
| --- | --- | --- |
| TCGA (n = 206) | 25 | 5 |
| MSK-2010 (n = 207) | 12 | 3 |
| MSK-IMPACT-2017 (n = 431) | 29 | 5 |
| Total # of patients = 844 | 66 (7.8%) | 13 (1.5%) |

**Figures:**

**Figure S1**


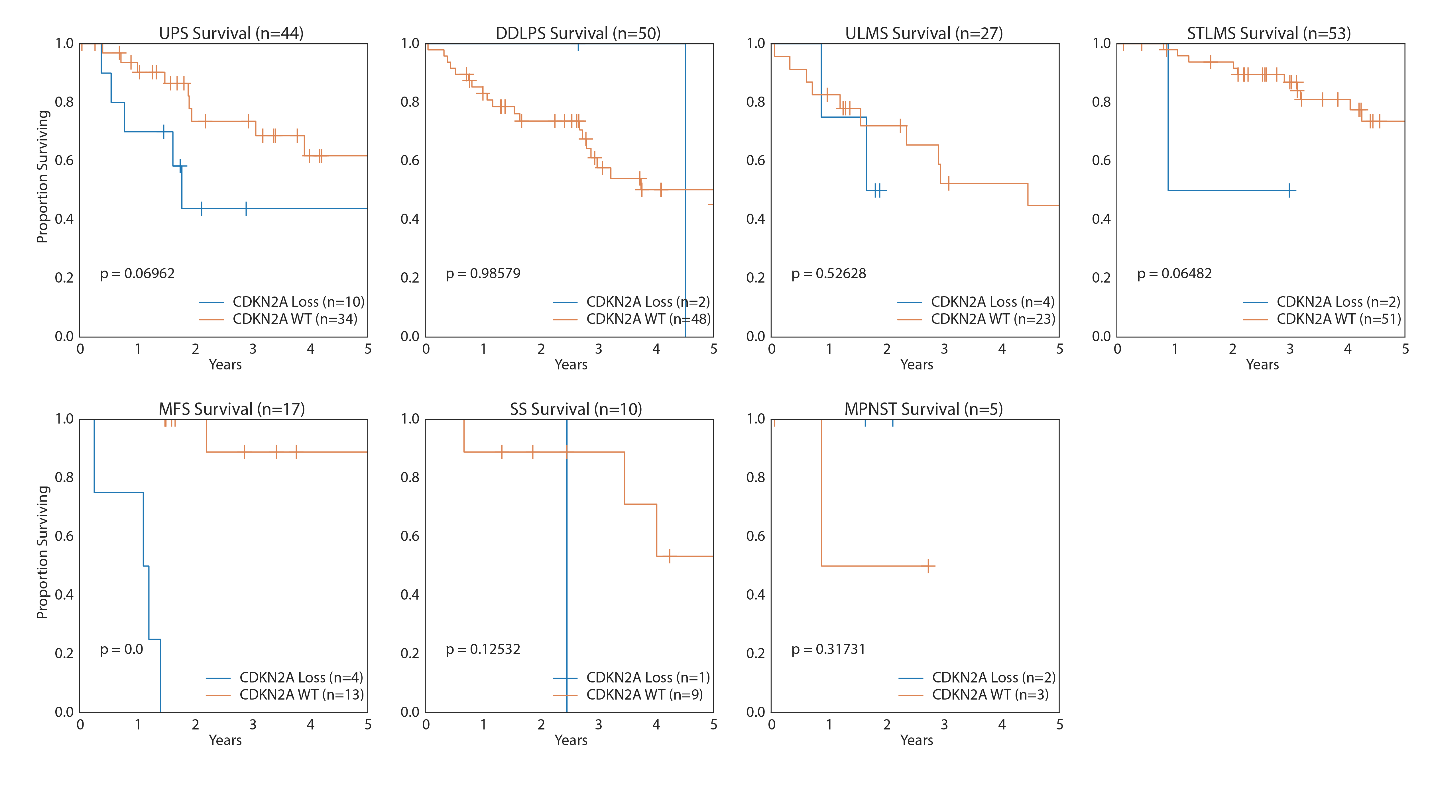


Survival plots for *CDKN2A* loss within histology reveals that poor prognosis appears to affect most histologies, most notably MFS (p=0), STLMS (p=0.06), UPS (p=0.07), and SS (p=0.12). Effect size and statistical significance is clearly affected by the small sample size of *CDKN2A* affected and total patients.

**Figure S2**


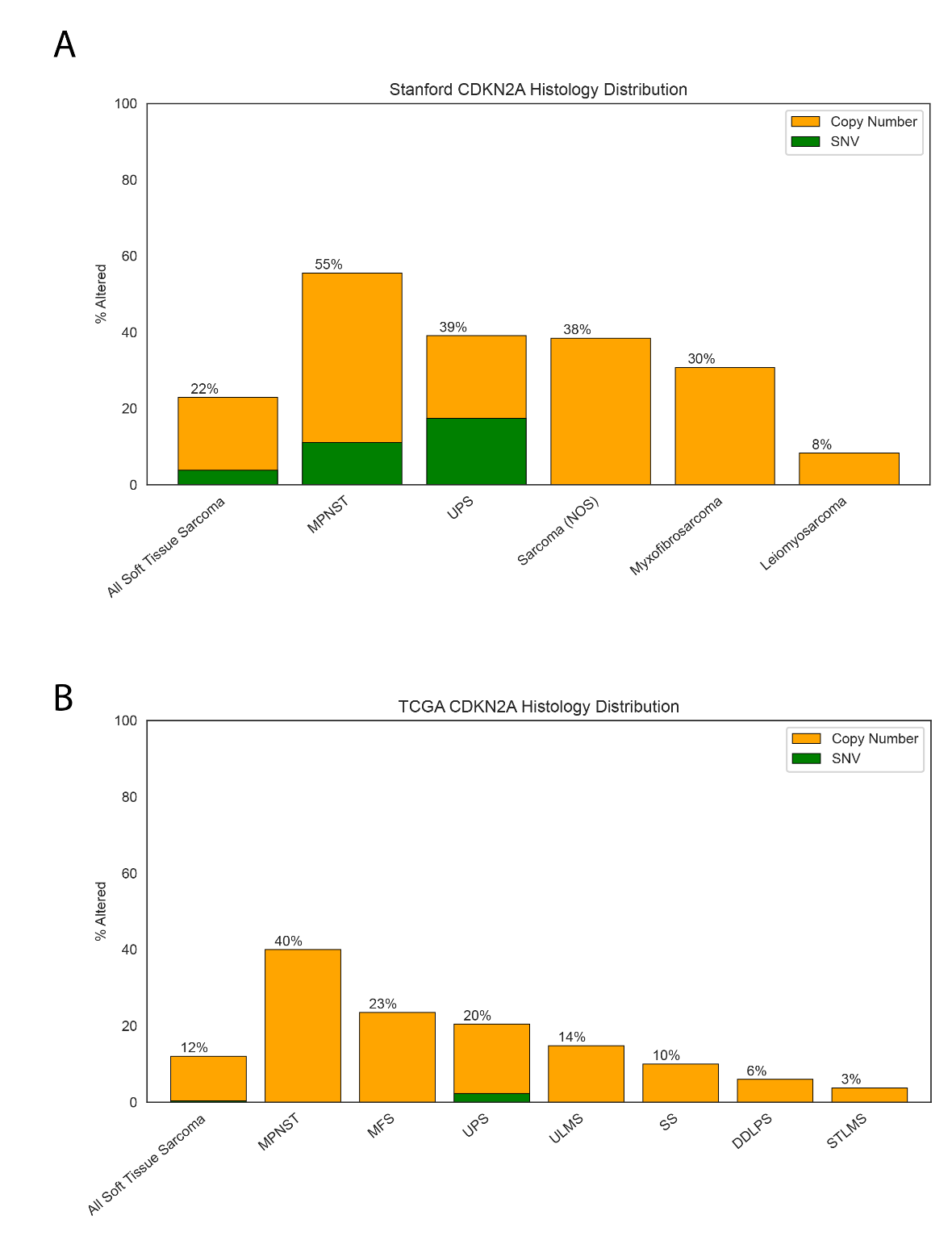


Frequency of *CDKN2A* mutations per histologic sarcoma type in **(A)** Stanford and **(B)** TCGA dataset. Copy number changes (orange) are much more prevalent than SNVs (green). Frequency counts are relatively similar to the Foundation Medicine database.
